# A Model Framework to Estimate Impact and Cost of Genetics-Based Sterile Insect Methods for Dengue Vector Control

**DOI:** 10.1371/journal.pone.0025384

**Published:** 2011-10-05

**Authors:** Nina Alphey, Luke Alphey, Michael B. Bonsall

**Affiliations:** 1 Mathematical Ecology Research Group, Department of Zoology, University of Oxford, Oxford, United Kingdom; 2 Oxitec, Limited, Oxford, United Kingdom; 3 Department of Zoology, University of Oxford, Oxford, United Kingdom; Universidade Federal do Rio de Janeiro, Brazil

## Abstract

Vector-borne diseases impose enormous health and economic burdens and additional methods to control vector populations are clearly needed. The Sterile Insect Technique (SIT) has been successful against agricultural pests, but is not in large-scale use for suppressing or eliminating mosquito populations. Genetic RIDL technology (Release of Insects carrying a Dominant Lethal) is a proposed modification that involves releasing insects that are homozygous for a repressible dominant lethal genetic construct rather than being sterilized by irradiation, and could potentially overcome some technical difficulties with the conventional SIT technology. Using the arboviral disease dengue as an example, we combine vector population dynamics and epidemiological models to explore the effect of a program of RIDL releases on disease transmission. We use these to derive a preliminary estimate of the potential cost-effectiveness of vector control by applying estimates of the costs of SIT. We predict that this genetic control strategy could eliminate dengue rapidly from a human community, and at lower expense (approximately US$ 2∼30 per case averted) than the direct and indirect costs of disease (mean US$ 86–190 per case of dengue). The theoretical framework has wider potential use; by appropriately adapting or replacing each component of the framework (entomological, epidemiological, vector control bio-economics and health economics), it could be applied to other vector-borne diseases or vector control strategies and extended to include other health interventions.

## Introduction

Around the world, vector-borne diseases cause medical and economic burdens as current control measures fail to cope, and face possible negative effects of local environmental change. There is a need to identify new or improved strategies that will remain effective despite growing insecticide and drug resistance [1]. Genetic techniques targeting insect vectors may provide new approaches to disease control; to illustrate their potential benefit, we focus on dengue, arguably the most important arboviral disease of humans.

Dengue is potentially severe, although it can present as a spectrum of symptoms from near-asymptomatic or mild febrile illness, through the classic incapacitating disease (seldom fatal), to its most severe forms dengue hemorrhagic fever (DHF) and dengue shock syndrome (DSS) [2]. Case fatality rates for DHF range from below 1% with modern intensive supportive therapy to over 20% without proper treatment, averaging 5% worldwide [3]. Dengue, the most rapidly spreading vector-borne disease in the tropics and subtropics, is endemic in over 100 (mainly low and middle income) countries and over 40% of the world's population live in areas at risk. An estimated 50–100 million infections occur annually; an annual average of over 0.9 million severe cases are reported to the World Health Organization (WHO) and roughly 18–19,000 dengue-related deaths are registered each year [2], [4], [5], [6]. All ages are affected, with most deaths and cases reported to WHO occurring among children (age 0–14 years) [4], [6].

Dengue is caused by any of four related flaviviruses. All four of these dengue virus serotypes are circulating in Asia, Africa and the Americas [5], though not all in all countries. Infection with any one serotype confers lifelong immunity to that type, and temporary cross-immunity to other serotypes [3], [7]. On subsequent heterotypic infection after cross-immunity wanes, antibody-dependent enhancement (ADE) increases the risk of more serious disease (and DHF) [3], [8]. Dengue is transmitted by the bite of an infective female mosquito; the main vector is the yellow fever mosquito *Aedes* (*Stegomyia*) *aegypti*, with the Asian tiger mosquito *Aedes albopictus* implicated as a secondary vector in some areas.

There are currently no drugs for dengue, and an effective and safe vaccine is unlikely to be available for several years [9], [10]. The *Aedes* vectors principally bite during daylight [11], and although preliminary evidence suggests that insecticide-treated bednets might provide some protection against dengue, studies with insecticide-treated materials (e.g. curtains, water container covers) have had mixed results [12], [13]. The only practical prevention method presently available is to control the principal vector. Current efforts focus on mosquito control and larval source reduction [4], [14], [15]. Complex urban settings present a major challenge for vector control activities, and in practice their effectiveness has been compromised due to issues of delivery, coverage and acceptability [2], [5], [16]. Dengue is a suitable target for genetic vector control strategies as it is specific to humans across most of its range (it has no significant animal reservoirs), has a single dominant vector, and area-wide vector control programs have in the past proven to be effective in controlling this disease, although those methods are now unacceptable or have not been sustainable [17], [18].

The Sterile Insect Technique (SIT) has been used successfully for suppressing or eliminating a number of agricultural pests [19], but there are no large-scale SIT programs in operation against any mosquito species. A number of trials were conducted against mosquitoes mostly in the 1960s and 1970s, and in recent years extensive research and development has been underway in preparation for commercial scale SIT [20], [21], [22]. Some technical difficulties with SIT using radiation-sterilization can potentially be overcome using genetically engineered strains, particularly loss of male fitness and absence of sex-separation mechanisms applicable on a large scale (which are necessary to release only males, as even sterile female mosquitoes bite) [21], [23], [24], [25]. Recently, *Ae. aegypti* mosquitoes suitable for use in the RIDL (Release of Insects Carrying a Dominant Lethal) system have been developed by transformation with repressible, dominant, late-acting, bisex-lethal genetic constructs (causing death after the effects of density-dependent larval mortality) [26]; such strains are the subject of this paper. This system has an in-built element of resistance dilution, which a theoretical study suggests can protect against the evolution of resistance to the genetic lethal construct [27].

An initial assessment of the potential impact of RIDL strategies on a generic mosquito-borne disease, using a simple mathematical model [28], suggested it is feasible to eliminate the disease from a human population of order 10^6^ in roughly one year. There is a trade-off between the time to virus eradication and the total number of mosquitoes released, which has economic implications. Numerous studies (see Supporting Information S1 & Table S3) report estimated costs of dengue in various countries from 1977 to 2005, mainly during epidemics, but these vary in details, methods, timing and the nature of costs included. Often these studies underestimate the true economic impact as they omit effects such as lost productivity, time off work or school, social disruption and lost tourism [2]. A prospective study [29] applying a common protocol in eight countries, is the most comprehensive study of dengue costs published to date and the first to develop comparable data over two hemispheres.

**Figure 1:**
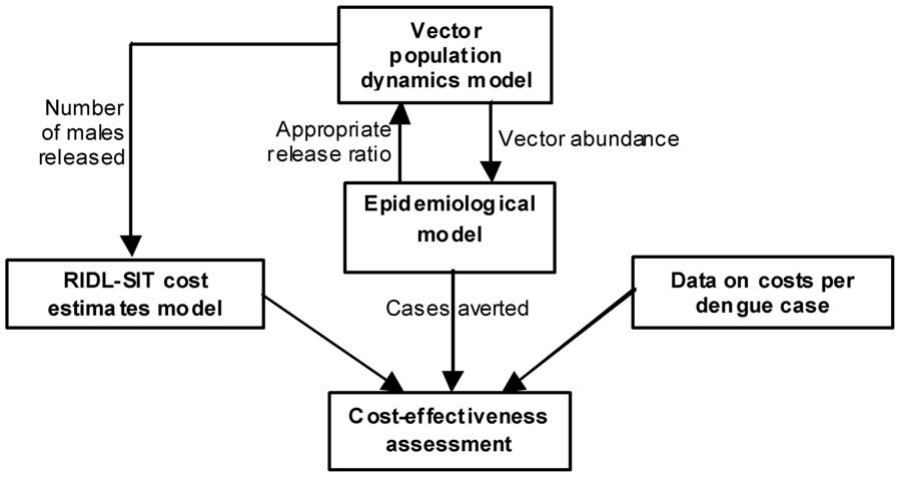
Overview of model components.

Dengue vector control in urban areas with current methods is costly [15]. Here we develop a combined model framework to analyze the approximate economic cost-effectiveness of genetic vector control (Figure 1). A mosquito population dynamics model predicts the population size of the vector (adult female mosquitoes). This is coupled with an epidemiological model to predict the disease dynamics, including the total number of dengue cases. This combined model captures key features, including density-dependent competition among *Ae. aegypti* larvae and the multi-annual cycles that are characteristic of dengue. It is not intended to be a comprehensive representation of the interaction between mosquito vectors, disease virus and humans, and we simplify the model components appropriately. For example, we ignore within-year seasonal fluctuations as modeling suggests these are unlikely to have a significant effect on multi-year patterns of incidence [30], we do not incorporate spatial heterogeneity, and we assume random mating among mosquitoes. We apply estimates of the costs of RIDL-based SIT for vector control, based on data for irradiation-based SIT, to investigate the potential cost-effectiveness of this strategy.

## Methods

The full derivation of the vector population dynamics and epidemiological models is set out in the Supporting Information S1. The state variables are listed in Table 1 and all parameters described in Table 2.

**Table 1:** State variables.

Vectors (adult female mosquitoes):	
*F*	total number of adult female vectors (susceptible, exposed and infectious)
*X*	susceptible
*Y_j_*	infectious with serotype *j*
**Hosts** (humans):	
*N*	total number of hosts (susceptible, exposed, infectious, cross-immune and recovered)
*S*	susceptible to all serotypes
*I_i_*	primary infection (infectious) with serotype *i*
*C_i_*	recovered from primary infection with serotype *i*, temporarily cross-immune to all other types
*R_i_*	recovered from primary infection with serotype *i*, susceptible to all other types
*I_ij_*	secondary infection with serotype *j,* following primary infection with serotype *i*
*R*	recovered from secondary infection, immune to all serotypes

**Table 2:** Parameters.

Symbol	Description	Default value	Range	Refs.
*σ*	Adult mosquito death rate (per day)	1/14 (chosen to be conservative)	1/15 to 1/3	[3], [30], [33], [55], [56], [57], [58], [59], [60]
*T*	Mosquito generation time (days), i.e. development period from egg to emerging adult	18.5	16.9–20.1	[3], [31], [32]
*E*	Daily egg production rate *per adult* mosquito (a female lays 2*E* per day on average, a male lays none)	8	7–9	[31], [32], [33]
*P*	The number of offspring produced by each adult per day that will survive to adulthood in the absence of density-dependent mortality (i.e. *E* adjusted for density-independent egg-to-adult survival)–estimated value, calculated using field data, depends on value of *σ* (*P/σ* is net reproductive rate)	0.7	0.2 to 0.7	[31], [32], [33]
*k*	Average number of vectors (adult female mosquitoes) per host (initial population *N*_0_)	2	0.3 to 20	[3], [11], [58], [61]
*α*	1/*α* is related to the number of breeding sites. Set α = log(*P/σ*)*/*[(2*kN*_0_ *E*)*^β^*] for given initial host population (*N*_0_)	≈1.5 ×10^−8^	≈4.5 ×10^−12^ to ≈1.0 ×10^−2^	[31]
*β*	Strength of larval density dependence	1	0.302 to 1.5	[31], [50]
*C*	Maintained ratio of RIDL males to pre-release equilibrium number of adult males (constant release policy)	10 or 1	various	N/A
*ν*	Human per capita birth rate (per day) Equal to human death rate	1*/*(60×365)	1*/*(60×365) to 1*/*(68×365)	N/A
*µ*	Human per capita death rate (per day) i.e. 60 year life span (default)	1*/*(60×365)	1*/*(60×365) to 1*/*(68×365) Thailand 1974–2010	[62], [63], [64]
*b*	Biting rate (number of bites per mosquito per day)	0.5	0.33 to 1	[58], [65]
*a*	Proportion of bites that successfully infect a susceptible human	0.38	0.25 to 0.75	[30], [66]
*c*	Proportion of bites that successfully infect a susceptible mosquito	0.38	0.20 to 0.75	[30], [66], [67]
*τ*	Virus latent period in humans (days) Intrinsic incubation period	5	3 to 12	[7], [68]
*ω*	Virus latent period in vectors (days) Extrinsic incubation period	10	7 to 14	[3], [7], [66], [68]
*γ*	Human recovery rate (per day) i.e. infectious period 6 days (default)	1*/*6	1*/*10 to 1*/*2	[3], [7], [29], [68]
*ψ*	Rate at which humans lose cross-immunity (per day) i.e. cross-immunity lasts 4 months (default)	1*/*(365×4*/*12)	1*/*(365×5*/*12) to 1*/*(365×2*/*12)	[3], [7], [30]
*χ*	Increased host susceptibility due to ADE	1.5	1 to 3	[3], [30]
*ζ*	(Alternative to *χ*) Increased transmissibility due to ADE	1	1	[3]
*ρ*	Proportion of hosts that recover from secondary infection (1-*ρ* die from DHF/DSS)	0.9999	1−0.05×(1−0.87) ≈ 0.9935 to 1	[3], [29], [37]

### Vector population dynamics model

We modify a simulation model for the population dynamics of *Ae. aegypti* developed by Dye [31], who used data from field studies [32], [33] and assessed the likely range of several parameters. Designating the number of vectors, i.e. female adult mosquitoes, at time *t* as *F*(*t*), prior to the start of releases we use a delayed differential equation comprising an adult mortality term and a term for adult emergence that incorporates both density-independent mortality of immature stages and a two-parameter function representing density-dependent larval mortality (equation S1). The number of vectors naturally settles at, or oscillates around, an equilibrium value 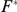
(equation S2; in deriving this we define *k* as the average number of vectors per host prior to control); if the numbers were perturbed they would increase (or decrease) back to equilibrium if density dependent competition among larvae were weak (i.e. low parameter 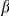 much smaller than 1), return to equilibrium overshooting back and forth on the way (damped oscillations with intermediate 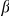) , or revert to oscillating around the equilibrium (strong density dependence, high 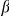). We modify equation S1 to incorporate the release, starting at time 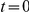, of homozygous male mosquitoes that carry a dominant, late-acting, bisex-lethal genetic construct (“RIDL males”, whose progeny die as late larvae after the effects of density-dependent larval mortality). Mass-reared adult RIDL males are released in quantities that maintain their numbers at a fixed ratio (the “release ratio” *C*) to the natural equilibrium number of adult males or females in the wild population, i.e. the total number of adult RIDL males 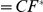 for 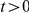. Only the larvae sired by wild type males survive to emerge as adults, so the adult emergence rate is proportionally reduced, assuming random mating.
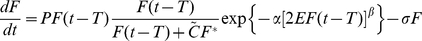
(1)


where 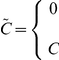 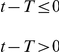


### Epidemiological model

In conjunction with the vector population dynamics model, we use a compartmental epidemiological model to describe the status of vectors and hosts with respect to the disease. Vectors, the adult female mosquitoes, are classed as susceptible (*X*) or infectious (*Y_i_*), and human hosts (*N*) are classed broadly as susceptible (*S* susceptible to all types, or *R_i_*, recovered from serotype *i* and susceptible to other types), infectious (*I_i_* primary infection, or *I_ji_* secondary infection with serotype *i* after past infection by type *j*), cross-immune (*C_i_*, not to be confused with *C*, the release ratio) or recovered (*R*). The intrinsic (in the host) and extrinsic (in the vector) incubation periods are represented using fixed duration time delays. The choice and range of values for entomological and epidemiological parameters is drawn from published literature (Table 2).

Full representation of all four dengue serotypes and the interactions between them requires a complicated mathematical model. To reduce the complexity we restrict the model to two serotypes. We label these *i*  = 1 and 2, which could be any pair of the four known serotypes. ADE manifests both as increased susceptibility to a secondary infection (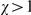), and as a risk of DHF or DSS from secondary infection, with a proportion (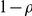) dying at the end of the infectious period. Although ADE may manifest as increased transmissibility from a host with a secondary infection (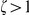), we do not use this alternative in our simulations (we keep 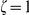); other authors found predictions from a similar model insensitive to whether ADE acts through transmissibility or susceptibility [30]. Together, equations 1 to 10 (given below) form a system of delay-differential equations representing the vector-pathogen-host dynamics.
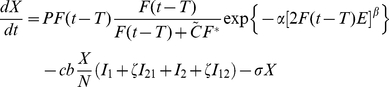
(2)

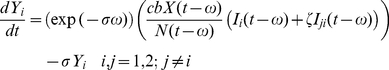
(3)

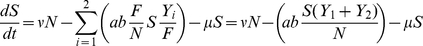
(4)

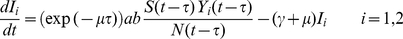
(5)

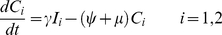
(6)

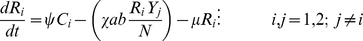
(7)

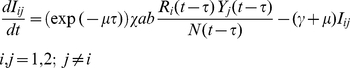
(8)

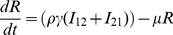
(9)

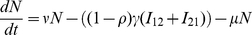
(10)


The two serotype model encapsulates the key properties of the disease dynamics. We use it to evaluate the RIDL genetic strategy for vector control by modeling the potential reduction in numbers of cases. Fewer cases will reduce the cost and burden of disease. The release ratio and duration of release of engineered male mosquitoes will drive the costs of the intervention.

We tested the sensitivity of our vector-epidemiological model to parameter values in the ranges shown in Table 2, for release ratio (*C*) 10, which is projected to achieve vector elimination, and initial human population 2 million (see Supporting Information S1).

### Estimating costs and assessing cost-effectiveness

The cost-effectiveness of any disease control strategy is the cost of achieving a specified outcome. For our study, we compare the projected costs per case averted by genetic vector control with estimates of the average cost of an episode of illness.

Most available cost information is already expressed in US dollars; where this is not so we convert using published exchange rates [34]. We inflate values to 2008 US dollars using GDP chained Price Index [35]. Drawing information from the available literature and various public sources, we identify SIT facility construction costs (Table S1) separately from operational costs (Table S2) where possible and attempt to fit regression curves to the available data for each, using a non-linear least-squares approach. This information is applied to generate a range of projected values for those two key broad elements of SIT cost. We use total cases, output from the simulation model, as our measure of the direct impact of the disease. The cost of dengue is estimated using a range of values for cost per case, based on published studies (Table S3). We also gather data on the per capita cost of conventional vector control methods (Table S4).

## Results

### Vector population dynamics

With our assumption that the vector population starts at, or oscillating around, its natural equilibrium 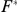, a sufficiently high release ratio (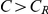 a threshold value) will eliminate the local vector population (*F* = 0), or a lower release ratio (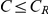) will suppress the vector population to a new, smaller, stable equilibrium, which decreases as *C* increases towards 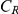. These results are independent of scale (i.e. size of host and vector populations); only the ratio of the post-release equilibrium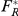 to the pre-release equilibrium 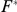 is relevant. With default parameter values (as in Table 2) the critical release ratio 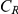 is approximately 1.19 (i.e. maintain 1.19 RIDL males for every wild type male at pre-release equilibrium), which will reduce the vector population to a new steady size a little over a third of its equilibrium before releases had started (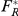 is approximately 0.34 times 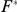).

### Epidemiology (no RIDL release)

The two serotype model exhibits characteristic epidemic cycles, with average period of c.5.5 years. (Four serotypes would be about 3.3–3.6 years per epidemic, in line with a 30-year four-serotype dataset from Thailand and a full four-serotype model [30]). Once the pattern is established, about 60% of the host population is recovered and immune to both serotypes.

The model can be analyzed in part by applying ecological and population biology techniques. For simplicity, we may assume the vector population is at equilibrium (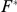), and the host birth (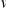) and death (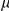) rates are equal and dengue-associated deaths are negligible (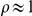) so that the host population (*N*) is constant. We can estimate the value of R_0_ inherent in our model from a dengue-naïve scenario, either from first principles or using mathematical methods (see Supporting Information S1):
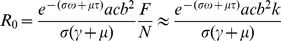
(11) With our default parameter values (Table 2), this estimated R_0_ is approximately 3.

With constant host population, we can assess the conditions under which dengue (one serotype) can invade naïve populations (where all hosts and vectors are susceptible to dengue, 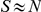 and 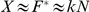, where *k* is the average number of vectors per host at equilibrium) by expressing equations 3 and 5 in the form of an invasion matrix (see Supporting Information S1). This method gives the entomological threshold for disease transmission, i.e. the minimum number of vectors (adult female mosquitoes) per host in a susceptible population that are necessary to sustain the disease, which we label 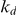:
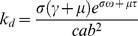
(12) With default parameter values (Table 2), 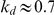. Simulations with a reasonable estimate of mortality from DHF/DSS (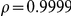, Table 2), confirm the entomological threshold is still approximately 0.7. The invasion threshold is where the basic reproduction number of dengue (R_0_) is equal to one [36].

These estimates for the basic reproduction number R_0_ and the entomological threshold *k_d_* are validated by comparison with other studies (Supporting Information S1).

### Control of dengue by release of RIDL mosquitoes

**Figure 2:**
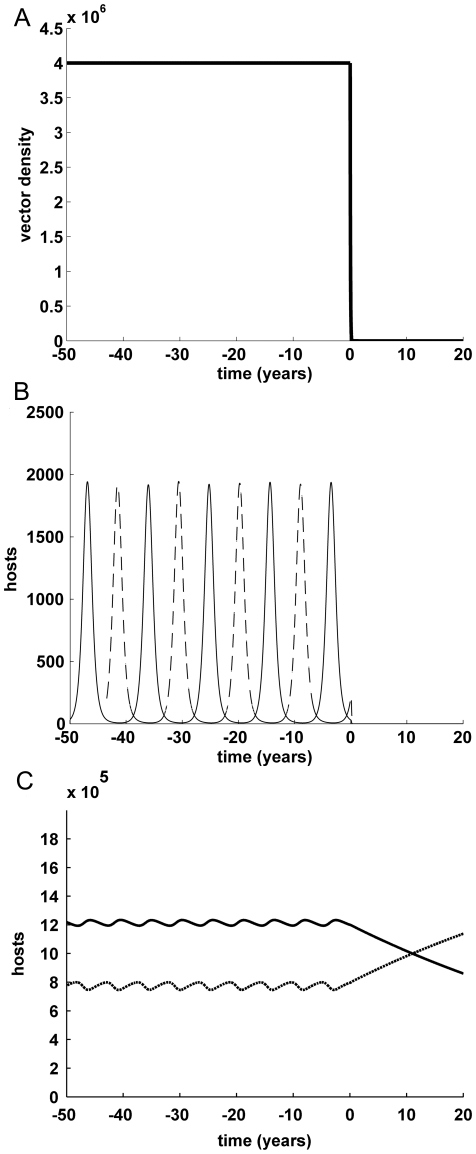
Vector and epidemiological dynamics, with release ratio 10∶1. Release ratio *C*: 10. Over time (A) total number of vectors, (B) total number of infectious hosts (primary or secondary infections), by serotype (1: solid line, 2: dashed), (C) total number of hosts recovered from secondary infection (solid) or susceptible to either or both serotypes (dashed). Default parameter values (Table 2), with initial conditions host population *N*_0_: 2 million and primary infections *I*_1_: 1, *I*_2_: 2. The release ratio is sufficiently high (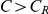), that the vector and virus are eliminated. Over subsequent years, immunity is lost from the host population.

**Figure 3:**
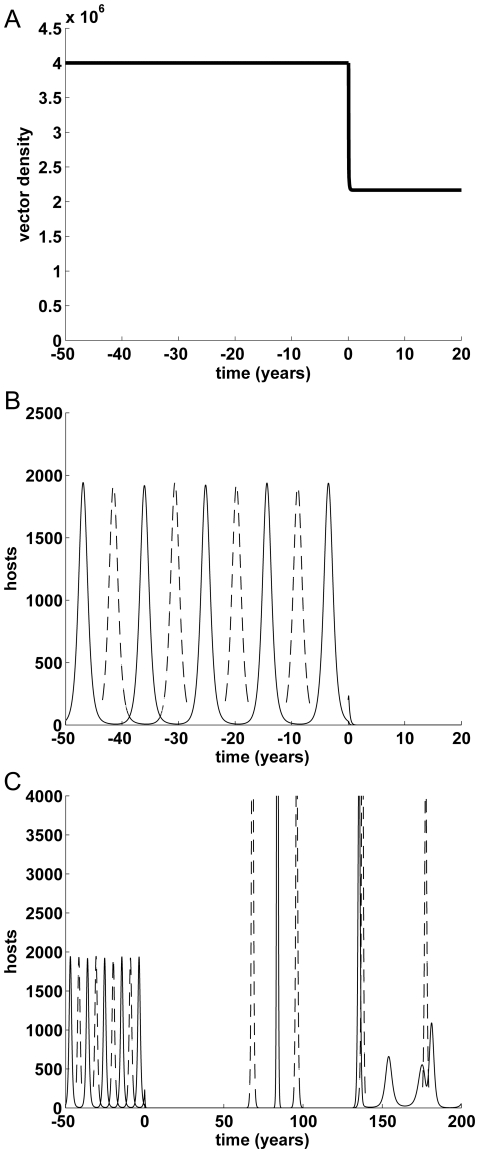
Vector and epidemiological dynamics, with release ratio 1∶1. Release ratio *C*: 1. Over time (A) total number of vectors, (B & C) total number of infectious hosts (primary or secondary infections), by serotype (1: solid line, 2: dashed); Default parameter values (Table 2), with initial conditions host population *N*_0_: 2 million and primary infections *I*_1_: 1, *I*_2_: 2. With this low release ratio (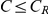), the vector population is reduced but remains above the transmission threshold vector abundance (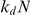); panel (C) is on different scales (note the much longer time period) and shows that the disease returns after initial suppression and persists in the longer term.

There are potentially three possible scenarios:

- with sufficiently high release ratio (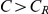), the vector and virus are eliminated (Figure 2);
- with intermediate release ratio (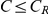), if the new equilibrium to which the vector population is reduced (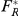) is below the threshold of vector abundance needed to sustain transmission (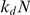), the virus is eliminated (equivalently, 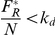 in terms of vectors per host);
- with too low release ratio (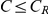), if the vector population is reduced but remains above the transmission threshold vector abundance (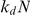) (i.e. vectors per host 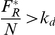), the virus may temporarily fade out but the disease persists in the longer term (Figure 3).

In practice, these thresholds (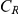 and 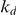) would be dynamic (e.g. due to immigration/emigration, fluctuating parameter values, and the effects of spatial heterogeneity), but these three potential outcomes would still apply for generically “sufficiently high”, “intermediate” or “too low” release ratios.

If the minimum possible non-zero equilibrium of vectors per host (with the value of 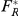 corresponding to critical release ratio *C_R_*) is more than 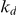, the intermediate scenario cannot occur so we could only eliminate the disease by eliminating the vector. This is the case with our default parameter values, under the assumptions of our models; in simulations a critical release ratio somewhere between 1.1 and 1.2 determines whether both or neither vector and virus are eliminated (this is comparable with the 1.19 calculated for the situation where there is no DHF/DSS mortality). However, with only 1 vector per host (*k*), and release ratio 1 (*C*), for example, the vector population is only reduced to 54% of its pre-release equilibrium value but this is enough to disrupt transmission of the disease and eliminate the virus.

Without eliminating the virus, it is possible to reduce the disease below detectable levels and sustain this by continued releases for more than 50 years with our default parameter values and release ratio 1 (Figure 3C). In our model this occurs as the numbers of infectious hosts and vectors fall to very small values before resurgence. In practice, the virus might temporarily fade out but be reintroduced by an infectious person or vector joining the population.

Entomological and epidemiological parameter values influence the transmission threshold number of vectors per host (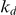) and are involved in determining whether there is any intermediate level of control that only suppresses the vector and yet eliminates the disease. In practice, it is likely that a control program would aim to eliminate the vector if that were feasible. Uncertainty in measuring parameters in the field, and deviations from model assumptions, make it wise to allow a margin of error when setting any release ratio.

We simulated the effect of RIDL male release in a dengue-endemic setting; because the simulated costs do not scale linearly with host or vector population sizes, we present results with initial human population size of either 2,000,000 (Figures 2 & 3) or 10,000. In terms of scale, these numbers of people might represent a city or small settlement, respectively, although our models do not reflect the different spatial heterogeneities and different scale of some implementation activities that would arise. With default parameter values, the number of infectious hosts peaks at below 10% of the host population (e.g. under 2,000 in the city, Figure 2B), with the mean proportion of infectious hosts just below half that (e.g. mean number 800 in the city setting), corresponding to disease incidence of under 2.5% per year on average (e.g. average 48–49,000 annual cases in a population of 2 million). We assessed the outcomes-the average number of infectious cases arising in a specified number of years from the start of RIDL releases and in the equivalent period with no releases (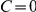) - for a program running for 5 or 10 years (Table 3), ignoring any residual effects beyond that period.

**Table 3:** Simulation results.

	Initial host population *N*_0_			
	2,000,000		10,000	
Release ratio *C*	1	10	1	10
Time to vector elimination (days)	N/A	285	N/A	211
Time to disease elimination (days)	N/A	176	N/A	107
Mean numbers of infectious hosts with no release program, *t*: 0 to +5 years	813	813	4	4
Mean numbers of infectious hosts with release program, *t*: 0 to +5 years	25	13	0	0
*Cases averted in 5 years* †	239,816	243,532	1,146	1,179
Mean numbers of infectious hosts with no release program, *t*: 0 to +10 years	815	815	4	4
Mean numbers of infectious hosts with release program, *t*: 0 to +10 years	13	6	0	0
*Cases averted in 10 years* †	487,947	491,664	2,370	2,403
RIDL males released (millions) initially (CF^*^)	4	40	0.02	0.2
RIDL males released (millions) per year (365σCF^*^)	104.3	1042.9	0.5	5.2
*Total released in 5 years (millions)*	525.4	5254.3	2.6	26.3
*Total released in 10 years (millions)*	1046.8	10468.6	5.2	52.3
RIDL males released each week per person	1.0	10.0	1.0	10.0

† The average duration of infection is 6 days (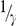), i.e. 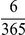 years, so an average of *n* infectious hosts at any time represents an average of 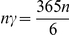
cases of infection per year. The cases averted are therefore calculated as ([mean no. with no releases–mean no. with releases]*/*average duration) × no. years.

As noted above, the high release ratio (*C* = 10) eliminates the vector and the virus, and although the low release ratio (*C* = 1) only suppresses the vector population and temporarily removes the disease, continuing releases at this low level gives protection for many years beyond the end of our assessment period (and beyond the practical duration of any genetic vector control program). If the disease is removed from (or barely present in) the host population, the herd immunity reduces over time as immune hosts die and are replaced by susceptible newborns (shown in Figure 2C). When this happens, the effective transmission threshold reduces over time (and the effective reproduction number R_t_ increases towards R_0_) and if the vector population has not been suppressed below the critical threshold for a susceptible population, the disease can eventually rebound strongly (Figure 3C); this is thought to have been a factor in the resurgence of dengue in Singapore despite strong vector control [18]. Assessed strictly over a 5 or 10 year time frame, the two release regimes have very similar effects and this is true in both settings. Where the vector is eliminated, this is projected to occur in under a year, and our model suggests the disease could be eliminated in three to six months. (The disease can be eliminated earlier than the vector, because this can occur soon after the vector falls below the transmission threshold.)

Production of RIDL males on this scale is potentially feasible, even applying release ratio 10 in a city of 2 million, which would require just over 1 billion RIDL males to be released annually over the duration of the program. For comparison, the largest SIT facility in the world, at El Pino in Guatemala, produces over 2 billion sterile male Mediterranean fruit flies per week [19], equivalent to over 100 billion annually.

### Estimated cost-effectiveness of genetic vector control

Using published data on SIT facility construction costs, we fitted models of construction cost of facility (including radiation source) against production capacity (see Supporting Information S1 & Table S1). For facility scale commensurate with our results above, our best fit was a rational function of the weekly production capacity (equation S8). There is no such discernable pattern to data on budgeted or actual operational or production costs for SIT facilities to relate the cost per million insects to the production quantity (see Supporting Information S1 & Table S2). We used the mean cost (US$813 per million insects), and a range from the minimum (US$172 for mosquito SIT in India in 1971) to the mean plus standard error of the data (US$813+819  =  US$1632).

Table 4 shows these projected costs applied to the simulated numbers of insects released and assessed against the simulated number of cases averted. Cases averted beyond the end of the program period are ignored. The unit cost is of the order of magnitude US$1 per 1000 insects in all scenarios simulated. The mean cost per case averted is roughly US$2–3 with release ratio 1 and $US20–30 with release ratio 10 (in health economic terms, these are average cost-effectiveness ratios, see points below on incremental cost-effectiveness). The mean cost per person protected per year during the assessment period is US$0.05–0.07 or US$0.52–0.68 (release ratio 1 or 10, respectively).

**Table 4:** Estimated cost of simulated releases.

	Initial host population *N*_0_			
	2,000,000		10,000	
Release ratio *C*	1	10	1	10
RIDL males released (millions) per week (7σCF^*^) = *production capacity* (*x*)	2.000	20.000	0.010	0.100
Construction costs 0.1297*x/*(1+0.0157*x*) (US$ millions)	0.252	1.974	0.001	0.013
**Five year program:**				
Total released in 5 years (*z*)	525.4	5254.3	2.6	26.3
Operational costs: mean 813*z*	0.427	4.272	0.002	0.021
Operational costs: range 172*z* to 1632*z*	0.090–0.857	0.904–8.575	0.000–0.004	0.005–0.013
Total (construction + operational) costs: mean	0.679	6.246	0.003	0.034
Total (construction + operational) costs: range	0.342–1.109	2.878–10.549	0.002–0.006	0.018–0.056
Cases averted in 5 years	239,816	243,532	1,146	1,179
Cost per case averted (mean)	US$2.83	US$25.65	US$3.00	US$29.11
Incremental cost-effectiveness ratio	N/A	US$1498	N/A	US$939
**Ten year program:**				
Total released in 10 years (*z*)	1046.8	10468.6	5.2	52.3
Operational costs: mean 813*z*	0.851	8.511	0.004	0.043
Operational costs: range 172*z* to 1632*z*	0.180–1.708	1.801–17.085	0.001–0.008	0.009–0.085
Total (construction + operational) costs: mean	1.103	10.485	0.005	0.056
Total (construction + operational) costs: range	0.432–1.960	3.775–19.059	0.002–0.009	0.022–0.098
Cases averted in 10 years	487,947	491,664	2,370	2,403
Cost per case averted (mean)	US$2.26	US$21.33	US$2.34	US$23.10
Incremental cost-effectiveness ratio	N/A	US$2524	N/A	US$1545

Numbers of insects released and cases averted are taken from Table 3. All costs are in 2008 US$ millions, except the mean costs per case averted and incremental cost-effectiveness ratio (which are in 2008 US$). The incremental cost-effectiveness ratio of 10∶1 release compared to 1∶1 release is the extra cost (total cost of the 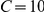 program minus that of the 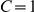 program) divided by the extra cases averted (cases averted with 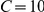 minus cases averted with 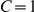).

**Figure 4:**
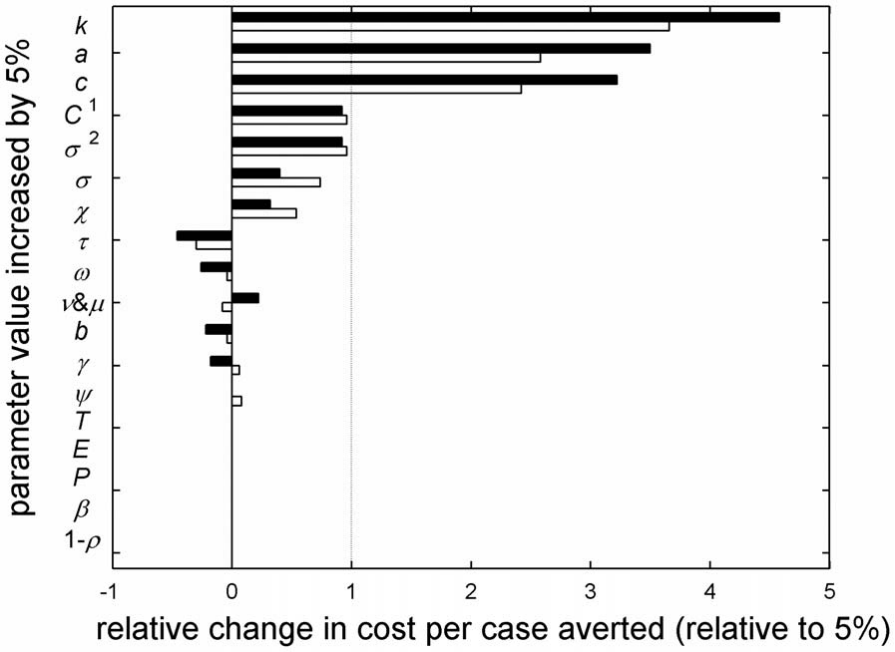
Importance of different parameter values to cost per dengue case averted. Relative change in cost per dengue case averted as a result of increasing each parameter value, one at a time, by 5%, for 5 year (black) or 10 year (white) release program. This is shown as “standard elasticity”, i.e. the relative change in the cost per case averted divided by the 5% relative change in each parameter value. Default parameter values (Table 2), initial host population *N*_0_: 2 million, and release ratio *C*: 10. ^1^ *C* was increased by 5% only in the calculations of cost, with the effective release ratio kept at 10 in the epidemiological model, to represent losses during delivery of engineered males. ^2^We also tested a 5% increase in the mosquito mortality rate 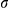 for males only, which affects the numbers to be released and hence the program costs.

Figure 4 shows the sensitivity of the costs per case averted to changes in parameter value estimates. The impact on that measure of outcome is generally smaller for the 10-year program as the increased construction cost, where a larger capacity facility is needed, is spread over a larger number of cases averted during the longer period. Increasing the number of vectors per host (*k*) has the largest effect, followed by the transmission rates in each direction (*a*, *c*). In all these three instances, the effect arises mainly because fewer cases are averted. These parameter value changes alter the epidemic pattern, shortening the period between epidemics. This change in timing means that without RIDL releases there are fewer infections in our reference period than our default scenario (fewer available to be averted by the intervention), and time 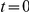 happens to coincide with the (higher) peak of infectious cases, consequently it takes two weeks longer to eliminate the virus. Higher transmission rates also decrease the entomological transmission threshold (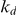), so the virus does not lose its potential to spread until the vector population reaches that lower level. Increasing the daily mortality rate of adult mosquitoes (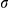) has only a small net effect. It increases the costs of RIDL releases, because the population of RIDL males must be replenished at a faster rate, which requires higher facility production capacity and output (this effect is also shown separately, and the relative change in outcome scaled to the relative change in parameter value is below 1 due to the economies of scale in SIT costs). Increasing 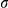 by 5% raises the entomological transmission threshold (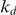) by about 9% (equation 12), so that the virus's reproduction number falls below one when the vector population is higher than in our default scenario and the virus is eliminated more quickly.

### Estimated benefits of reducing the burden of disease

Table S3 shows the range of dengue costs reported in various studies (see Supporting Information S1). Several of these underestimate the cost per case of illness–and therefore of the potential value of the benefit of reducing the number of cases–as they only include hospitalized cases, define costs narrowly and/or adopt a patient not societal perspective. A recent, comprehensive and consistent, prospective study across eight countries into the costs of dengue [29], estimated average costs per clinically ill case of US$ 357 (Asia) to US$793 (Americas). This study distinguished between ambulatory and hospitalized cases, and included direct medical costs, direct non-medical costs (such as transportation, food and lodging associated with seeking or obtaining medical care) and some indirect costs (days of school or paid work lost by the patient or a household member providing care during the illness episode). The lowest societal perspective cost reported is US$59 for the 1994 epidemic in Nicaragua [15].

The costs apply mainly to cases that are clinically ill. About 76% of dengue infections are thought to be asymptomatic or mild, and 24% of cases are clinically ill [37], although the few long-term prospective cohort studies from 1980 to the present report ratios of apparent cases to inapparent infections (without clinical signs) at 1∶0.9 to 1∶13 [38], [39], [40], [41], [42] and other reports range from 1∶60 to 1.7∶1 [3]. If only 24% of the infectious hosts simulated in our model incur these costs of illness and the rest incur no cost, the overall average cost per case (of any severity) is US$86–190 (based on US$357–793 per clinically ill case), or perhaps as little as US$14.16 (based on US$59). These all substantially exceed the US$2–3 average cost of RIDL-based vector control per case averted assuming the lower release ratio (*C* = 1) and the likely range significantly exceeds the US$20–30 average cost per case averted with high release ratio (*C* = 10).

As well as the average cost-effectiveness values discussed above, it is appropriate to consider the incremental cost-effectiveness ratio (ICER) of a program with release ratio 10 compared with a program with release ratio 1. This indicator reflects the marginal costs of the additional insects released and the extra benefit obtained by doing so. The ICERs shown in Table 4 range from US$939 to US$2524, which exceed the overall average societal cost per infection US$86–190, so using our measure (cases averted during the 5 or 10 year period) the extra cost of the higher release ratio is not worth the additional health effects obtained by disrupting virus transmission more quickly. In all four illustrated scenarios, the release ratio 1 program is preferred.

A significant reduction of dengue prevalence, or local elimination, might be expected to result in much lower conventional mosquito control costs (and remaining costs such as some surveillance are already included in our SIT cost estimates). Using data on the cost of vector control measures (e.g. spraying adulticides, larviciding, in some cases including costs such as packing, transport, training and supervision) of varying effectiveness, in a range of countries (Table S4), we estimate a mean annual per capita cost of $0.765. Using this value, annual vector control costs in the example of our simulated city of 2 million people would be US$1.53 million and for 10,000 people would be US$7,650. A major proportion of this would likely be saved, in addition to the direct and indirect costs of illness shown above, but the amount cannot be quantified from the available data.

## Discussion

We have shown by mathematical modeling (subject to caveats and model limitations discussed below) that genetic control of the dengue vector, using RIDL male releases in a SIT program, can eliminate the disease at a lower cost than the direct and indirect cost savings from the illnesses averted. Our model suggests this disease can effectively be removed from the population in a time scale of the order of six months or less. This can be achieved even where the vector is not suppressed to a level below the entomological threshold for disease transmission, provided there is sufficient disruption to transmission. Continuing the program of releases can provide effective protection for many years beyond the five or ten year assessment period. Wider benefits to the economy (such as future productivity gains resulting from lives saved, or increased tourism revenues) will clearly arise but have not been quantified.

We have assumed that the genetic construct is 100% lethal and that released RIDL males are fully competitive and available to take part in mating. Generally, of all transgenic insertion lines created, only those with sufficiently close to 100% lethality in heterozygous form are chosen for further development; those undergo tests for traits such as mating competitiveness, and only successful lines progress through the phases. In diverse species, lethality achieved by a single copy of a bisex-lethal or female-lethal RIDL construct has been 97–100% [26], [43], [44], [45]. The OX513A bisex-lethal RIDL strain of *Aedes aegypti* (first described in [26]), which is a lead candidate for field implementation, appeared to be fully competitive in trials under semi-field conditions in Malaysia [46]. Similar results have been achieved with different constructs in other species; one transgenic line of Mediterranean fruit fly *Ceratitis capitata* exhibits 100% embryonic lethality and is described as highly competitive with wild type Medfly in laboratory and field cage tests [47]. For field use, the mosquito strain to be released is made more similar to the local population by introgressing the transgene into the appropriate genetic background (OX513A exists in several genetic backgrounds). Although reductions in lethality and mating competitiveness are expected to be low, losses during handling and distribution might be more significant; any of these would require a reciprocal increase in numbers released, with consequent increase in cost. We do not specify how RIDL males might be distributed so as to be available to mate essentially at random with females in the wild population. The method(s) of delivery would necessarily need to be designed to suit any particular location. In cities there typically exists easy road access on a fine scale, so distribution using vehicles could be straightforward. Agricultural SIT programs often use aerial release, sometimes with GPS guidance systems, requiring expensive equipment and specialist personnel.

Like all models, our combined framework necessarily reflects a simplified version of reality and our results should not be interpreted as accurate predictions of outcomes in any specific setting. Features such as seasonal variations might need to be incorporated to obtain a reasonable fit to the incidence, epidemic peaks and multi-annual period of dengue if validating against a particular dataset. Our models capture the most essential characteristics of mosquito population and dengue dynamics. The growing body of literature on dengue is identifying further features that appear also to be important, such as heterogeneities in mosquito biting behavior, the contribution of human movement to the spread of dengue, and the spatially and temporally focal nature of transmission [48], [49]. We are also constrained by the availability of data. The characterization of density-dependent competition among *Aedes aegypti* larvae is better than for other mosquito species, but is based on a limited data set and subject to the assumptions under which Dye's model was statistically fitted to that data [50]. Refined cost estimates specific to the RIDL technology are not yet available. Our cost model uses data for conventional SIT primarily against agricultural pests. This will probably overestimate RIDL-based SIT costs because the larger agricultural insect species and use of dyes as markers both produce more waste material (which is expensive to dispose of), and the cost to purchase, transport, operate and keep secure a radiation source would not be incurred by a genetic control program. Conversely, the information often excludes or is unclear on costs of distributing the released insects (see Supporting Information S1) so our formula will underestimate operational costs in that respect. Our costs will also be overstated because we have not accounted for any reduction in conventional vector control costs, because of lack of detailed information.

In keeping the model relatively simple, we have ignored other possible epidemiological benefits. We assumed a constant case fatality rate, but this depends on the quality of care and the stage at which the patient is admitted to hospital. Hospitals can be overwhelmed during periods of intense epidemic transmission (for example, in April 2008 military support was called on to expand care beyond beleaguered public hospitals in Rio de Janeiro, Brazil) and patient management may suffer at such times. By reducing the number of hospital admissions during peak dengue periods, the vector control strategy might also decrease case fatality rates and thus save further lives. The sheer scale of an epidemic can also have implications for other health costs and services; for example, the dengue outbreak in North Queensland, Australia, in 2008/9 led to shortages of blood supplies at blood donor centers [51].

The undiscounted cost per case averted during a fixed period is a crude measure of cost-effectiveness of a health intervention. The World Development Report “Investing in Health” [52] makes extensive use of the Disability Adjusted Life Year (DALY) lost, a key measure of the burden of disease, to judge which health improving interventions deserve the highest priority for public action. The DALY reflects the total amount of healthy life lost to premature mortality or disability, taking into account how far into the future the loss occurs and weighting the relative importance of healthy life at different ages [53]. Eliminating the disease averts all the lost DALYs. For an assessment quantifying the DALYS gained (including beyond the end of the control program), either the model would have to be expanded to include the age structure of the host population or a reasonable estimate of stable age distribution at onset would be needed. Also, the amount and timing of cash flows associated with program costs should be discounted to present values using an appropriate risk-adjusted discount rate.

More sophisticated model components could bring in other elements, such as spatial structure in human and vector populations, variation in biting rates, seasonal fluctuations, or changes in host behavior (e.g. relaxing control efforts), according to what were deemed key features for the purpose of the question to be addressed. Better data would be needed to parameterize and validate such models properly, and the additional detail would potentially affect more than one component of the framework; for example, good information about vector populations over space and time requires adequate entomological surveillance and those monitoring activities will have associated costs.

Extension of our analysis could be customized to relevant country- or region-specific epidemiological, entomological, healthcare and economic data. Appropriate scenarios could be simulated, detailed estimates of constructing and operating a sterile mosquito release program could be prepared for a production facility of appropriate size and location, the projected DALYs saved could be valued at the per capita Gross National Income, and the effect on the wider impact of dengue on that economy could be assessed. In practice, such a wealth of specific data is unlikely to be available and an accurate fully inclusive cost-benefit analysis seems infeasible at least in the shorter term. However, preliminary approaches using available data could be used to identify the parameter values and other estimates to which the conclusions are most sensitive and to direct experimental, ecological, economic and other studies towards filling those key knowledge gaps.

The study presented here indicates that this genetic vector control strategy has the potential to be a cost-effective intervention. Deterministic models provide an appropriate framework for the purpose of preliminary assessment, and predictions from these models demonstrate the importance of key parameters (such as the mean number of vectors per host, which directly affects release numbers and hence costs). This information is likely to be highly relevant for future analysis. For example, in practice the actual numbers of RIDL males released and the release interval are likely to vary around planned values because of production variation, operational management, labor and equipment capacity, the weather and other factors. Prospective stakeholders will be interested in the chance of failure due to such fluctuations, and what planned capacity, release rate and release interval would be required to give at least a specified chance (e.g., 95%) of achieving a particular objective (e.g., no reported locally transmitted dengue infections) by a target date. Such questions could be investigated using a risk-modeling simulation approach that builds on the understanding gained from the present study.

The framework demonstrated here could be adapted to apply to other diseases (or combination of diseases vectored by the same species), other scenarios (such as including interactions with a vaccine or other interventions), or variant or alternative genetic technologies (for example, a program releasing *Ae. aegypti* RIDL eggs into natural breeding sites, or strategies aimed at converting the vector population to insects that are refractory to disease transmission).

Although there has been a focus on economic arguments for control of vector-borne diseases, there are wider potential benefits to society, such as environmental improvements from reduced insecticide use. Funding, technical and other support from governments and international agencies is likely to be prominent in any successful large-scale elimination of vector-borne disease. Tackling infectious diseases relates to several of the UN's Millennium Development Goals, to reduce child mortality, improve maternal health, combat diseases such as malaria, eradicate extreme poverty and hunger, and achieve universal primary education [54]. The control of diseases such as dengue and malaria is a humanitarian goal that addresses the basic human right to a healthy life.

## Supporting Information

Supporting Information S1 Models: vector population dynamics model, epidemiological model; Sensitivity of combined epidemiological model to parameter values; Determining basic reproduction number and entomological threshold (invasion analysis); Validating basic reproduction number and transmission threshold; Estimated cost-effectiveness of genetic vector control; References.
(DOC)

Table S1 SIT facility construction costs.
(DOC)

Table S2 SIT facility production or operational costs.
(DOC)

Table S3 Estimated costs per dengue case.
(DOC)

Table S4 Estimated per capita spending on vector control.
(DOC)

## References

[1] Sachs J, Malaney P (2002). The economic and social burden of malaria.. Nature.

[2] Gubler DJ (2002). Epidemic dengue/dengue hemorrhagic fever as a public health, social and economic problem in the 21st century.. Trends in Microbiology.

[3] Gubler DJ, Kuno G (1997). Dengue and dengue hemorrhagic fever..

[4] WHO (2009). http://www.who.int/mediacentre/factsheets/fs117/en/.

[5] WHO-TDR (2007). Report of the Scientific Working Group on Dengue, 2006. Geneva, Switzerland: Special Programme for Research & Training in Tropical Diseases (TDR)/World Health Organization.. TDR/SWG/.

[6] WHO (2008). http://www.who.int/healthinfo/global_burden_disease/GBD_report_2004update_AnnexA.pdf.

[7] Sabin AB (1952). Research on Dengue during World War II.. Am J Trop Med Hyg.

[8] Sangkawibha N, Rojanasuphot S, Ahandrik S, Viriyapongse S, Jatanasen S (1984). Risk Factors in Dengue Shock Syndrome: A prospective epidemiologic study in Rayong, Thailand: I. the 1980 outbreak.. American Journal of Epidemiology.

[9] Durbin AP, Whitehead SS, Rothman AL (2010). Dengue Vaccine Candidates in Development.. Dengue Virus.

[10] Zorlu G, Fleck F (2011). Dengue vaccine roll-out: getting ahead of the game.. Bulletin of the World Health Organization.

[11] Yasuno M, Tonn R (1970). A study of biting habits of *Aedes aegypti* in Bangkok, Thailand.. Bulletin of the World Health Organization.

[12] Lenhart A, Orelus N, Maskill R, Alexander N, Streit T (2008). Insecticide-treated bednets to control dengue vectors: preliminary evidence from a controlled trial in Haiti.. Tropical Medicine & International Health.

[13] Lenhart A, Castillo CE, Trongtokit Y, Villegas E, Apiwathnasorn C (2010). Cluster randomized trials of insecticide treated materials (ITMs) for dengue vector control in Latin America and SE Asia..

[14] Clark DV, Mammen MP, Nisalak A, Puthimethee V, Endy TP (2005). Economic impact of dengue fever/dengue hemorrhagic fever in Thailand at the family and population levels.. American Journal of Tropical Medicine and Hygiene.

[15] Torres JR, Castro J (2007). The health and economic impact of dengue in Latin America.. Cad Saúde Pública.

[16] Siqueira JB, Jr, Martelli CMT, Coelho GE, Da Rocha Simplício AC, Hatch DL (2005). Dengue and dengue hemorrhagic fever, Brazil, 1981-2002.. Emerging Infectious Diseases.

[17] Slosek J (1986). *Aedes aegypti* mosquitoes in the Americas: A review of their interactions with the human population.. Social Science & Medicine.

[18] Ooi E-E, Goh K-T, Gubler DJ (2006). Dengue prevention and 35 years of vector control in Singapore.. Emerging Infectious Diseases.

[19] Dyck VA, Hendrichs J, Robinson AS (2005). Sterile Insect Technique: Principles and practice in Area-Wide Integrated Pest Management..

[20] Asman SM, McDonald PT, Prout T (1981). Field studies of genetic control systems for mosquitoes.. Annual Review of Entomology.

[21] Benedict MQ, Robinson AS (2003). The first releases of transgenic mosquitoes: an argument for the sterile insect technique.. Trends Parasitol.

[22] IAEA (2008). Model Business Plan for a Sterile Insect Production Facility..

[23] Alphey L, Nimmo DD, O'Connell S, Alphey N, Aksoy S (2007). Insect population suppression using engineered insects.. Transgenesis and the management of vector-borne disease.

[24] Fu G, Lees RS, Nimmo D, Aw D, Jin L (2010). Female-specific flightless phenotype for mosquito control.. Proceedings of the National Academy of Sciences USA.

[25] Alphey L, Benedict M, Bellini R, Clark GG, Dame DA (2010). Sterile-Insect Methods for Control of Mosquito-Borne Diseases: An Analysis.. Vector-Borne and Zoonotic Diseases.

[26] Phuc HK, Andreasen MH, Burton RS, Vass C, Epton MJ (2007). Late-acting dominant lethal genetic systems and mosquito control.. BMC Biology.

[27] Alphey N, Bonsall MB, Alphey L (2011). Modeling resistance to genetic control of insects.. Journal of Theoretical Biology.

[28] Atkinson MP, Su Z, Alphey N, Alphey LS, Coleman PG (2007). Analyzing the control of mosquito-borne diseases by a dominant lethal genetic system.. Proceedings of the National Academy of Sciences of the United States of America.

[29] Suaya JA, Shepard DS, Siqueira JB, Martelli CT, Lum LCS (2009). Cost of Dengue Cases in Eight Countries in the Americas and Asia: A Prospective Study.. American Journal of Tropical Medicine and Hygiene.

[30] Wearing HJ, Rohani P (2006). Ecological and immunological determinants of dengue epidemics.. Proceedings of the National Academy of Sciences of the United States of America.

[31] Dye C (1984). Models for the population dynamics of the yellow fever mosquito, *Aedes aegypti*.. Journal of Animal Ecology.

[32] Southwood TRE, Murdie G, Yasuno M, Tonn RJ, Reader PM (1972). Studies on the life budget of *Aedes aegypti* in Wat Samphaya, Bangkok, Thailand.. Bulletin of the World Health Organization.

[33] Sheppard PM, Macdonald WW, Tonn RJ, Grab B (1969). The Dynamics of an Adult Population of *Aedes aegypti* in Relation to Dengue Haemorrhagic Fever in Bangkok.. Journal of Animal Ecology.

[34] US Federal Reserve (2009). http://federalreserve.gov/releases/h10/Hist/.

[35] US Office of Management and Budget (2008). Budget of the United States Government: Historical Tables Fiscal Year 2009.. http://www.gpoaccess.gov/usbudget/fy09/hist.html.

[36] Fine PEM. Population biology of infectious diseases: The control of infectious disease.. Anderson RM, May RM, editors; 1982 March 14-19; Berlin.

[37] Shepard DS, Suaya JA, Halstead SB, Nathan MB, Gubler DJ (2004). Cost-effectiveness of a pediatric dengue vaccine.. Vaccine.

[38] Balmaseda A, Hammond SN, Tellez Y, Imhoff L, Rodriguez Y (2006). High seroprevalence of antibodies against dengue virus in a prospective study of schoolchildren in Managua, Nicaragua.. Tropical Medicine & International Health.

[39] Burke DS, Nisalak A, Johnson DE, Scott RM (1988). A Prospective Study of Dengue Infections in Bangkok.. American Journal of Tropical Medicine and Hygiene.

[40] Endy TP, Chunsuttiwat S, Nisalak A, Libraty DH, Green S (2002). Epidemiology of Inapparent and Symptomatic Acute Dengue Virus Infection: A Prospective Study of Primary School Children in Kamphaeng Phet, Thailand.. American Journal of Epidemiology.

[41] Endy TP, Yoon I-K, Mammen MP (2010). Prospective Cohort Studies of Dengue Viral Transmission and Severity of Disease. In: Rothman AL, editor. Dengue Virus.. Berlin Heidelberg: Springer.

[42] Porter KR, Beckett CG, Kosasih H, Tan RI, Alisjahbana B (2005). Epidemiology Of Dengue And Dengue Hemorrhagic Fever In A Cohort Of Adults Living In Bandung, West Java, Indonesia.. American Journal of Tropical Medicine and Hygiene.

[43] Fu G, Condon KC, Epton MJ, Gong P, Jin L (2007). Female-specific insect lethality engineered using alternative splicing.. Nature Biotechnology.

[44] Gong P, Epton MJ, Fu G, Scaife S, Hiscox A (2005). A dominant lethal genetic system for autocidal control of the Mediterranean fruitfly.. Nature Biotechnology.

[45] Stainton KC, Condon GC, Fu G, O'Connell S, Alphey L (unpublished data) Development of a Female-Lethal Genetic System in the Mexican Fruit Fly, *Anastrepha ludens*.

[46] Lee H, Vasan S, Nazni W, Shanaz M (2008). Scientific Report on the Innovative Application of *Aedes aegypti* RIDL-Sterile Insect Technique to Combat Dengue and Chikungunya in Malaysia..

[47] Schetelig MF, Caceres C, Zacharopoulou A, Franz G, Wimmer EA (2009). Conditional embryonic lethality to improve the sterile insect technique in *Ceratitis capitata* (Diptera: Tephritidae).. BMC Biology.

[48] Stoddard ST, Morrison AC, Vazquez-Prokopec GM, Paz Soldan V, Kochel TJ (2009). The Role of Human Movement in the Transmission of Vector-Borne Pathogens.. PLoS Neglected Tropical Diseases.

[49] Mammen MP, Pimgate C, Koenraadt CJM, Rothman AL, Aldstadt J (2008). Spatial and Temporal Clustering of Dengue Virus Transmission in Thai Villages.. PLoS Medicine.

[50] Legros M, Lloyd AL, Huang Y, Gould F (2009). Density-Dependent Intraspecific Competition in the Larval Stage of *Aedes aegypti* (Diptera: Culicidae): Revisiting the Current Paradigm.. Journal of Medical Entomology.

[51] Wight A (2009). Thirst for blood grows as dengue dries donations..

[52] World Bank (1993). World Development Report 1993: Investing in Health..

[53] Murray CJL (1994). Quantifying the burden of disease: The technical basis for disability-adjusted life years.. Bulletin of the World Health Organization.

[54] May RM (2007). Parasites, people and policy: infectious diseases and the Millennium Development Goals.. Trends in Ecology and Evolution.

[55] Day JF, Edman JD, Scott TW (1994). Reproductive Fitness and Survivorship of *Aedes aegypti* (Diptera: Culicidae) Maintained on Blood, with Field Observations from Thailand.. Journal of Medical Entomology.

[56] Harrington LC, Buonaccorsi JP, Edman JD, Costero A, Kittayapong P (2001). Analysis of Survival of Young and Old *Aedes aegypti* (Diptera: Culicidae) from Puerto Rico and Thailand.. Journal of Medical Entomology.

[57] Muir LE, Kay BH (1998). *Aedes aegypti* survival and dispersal estimated by mark-release-recapture in northern Australia.. American Journal of Tropical Medicine and Hygiene.

[58] Trpis M, Hausermann W (1986). Dispersal and other Population Parameters of *Aedes aegypti* in an African Village and their Possible Significance in Epidemiology of Vector-Borne Diseases.. American Journal of Tropical Medicine and Hygiene.

[59] Trpis M, Hausermann W, Craig GB (1995). Estimates of Population Size, Dispersal, and Longevity of Domestic *Aedes aegypti aegypti* (Diptera: Culicidae) by Mark-Release-Recapture in the Village of Shauri Moyo in Eastern Kenya.. Journal of Medical Entomology.

[60] Maciel-De-Freitas R, Codeco CT, Lourenco-De-Oliveira R (2007). Body size-associated survival and dispersal rates of *Aedes aegypti* in Rio de Janeiro.. Medical and Veterinary Entomology.

[61] Jeffery JAL, Thi Yen N, Nam VS, Nghia LT, Hoffmann AA (2009). Characterizing the *Aedes aegypti* Population in a Vietnamese Village in Preparation for a *Wolbachia*-Based Mosquito Control Strategy to Eliminate Dengue.. PLoS Neglected Tropical Diseases.

[62] National Statistical Office of Thailand (1996). The 1995-1996 Survey of Population Change: Table 5 Expectation of Life at Birth.. http://web.nso.go.th/en/survey/popchan/popchgt5.htm.

[63] United Nations Population Division (2006). World Mortality Report 2005.. http://www.un.org/esa/population/publications/worldmortality/WMR2005.pdf.

[64] United Nations Population Division (2009). World Population Prospects: The 2008 Revision - Thailand country profile.. http://esa.un.org/unpp/p2k0data.asp.

[65] Scott TW, Chow E, Strickman D, Kittayapong P, Wirtz RA (1993). Blood-Feeding Patterns of *Aedes aegypti* (Diptera: Culicidae) Collected in a Rural Thai Village.. Journal of Medical Entomology.

[66] Watts DM, Burke DS, Harrison BA, Whitmire RE, Nisalak A (1987). Effect of Temperature on the Vector Efficiency of *Aedes aegypti* for Dengue 2 Virus.. American Journal Tropical Medicine and Hygiene.

[67] Schneider JR, Mori A, Romero-Severson J, Chadee DD, Severson DW (2007). Investigations of dengue-2 susceptibility and body size among *Aedes aegypti* populations.. Medical and Veterinary Entomology.

[68] Siler JF, Hall MW, Hitchens AP (1926). Dengue: its history, epidemiology, mechanism of transmission, etiology, clinical manifestations, immunity, and prevention.. Philippine Journal of Science.

