# Supplementary material for: A Model Framework to Estimate Impact and Cost of Genetics-Based Sterile Insect Methods for Dengue Vector Control
Source: PLoS One. 2011 Oct 5;6(10):e25384. doi: 10.1371/journal.pone.0025384 (PMC3187769; doi:10.1371/journal.pone.0025384)
Supplement: Supporting Information S1 — Models: vector population dynamics model, epidemiological model; Sensitivity of combined epidemiological model to parameter values; Determining basic reproduction number and entomological threshold (invasion analysis); Validating basic reproduction number and transmission threshold; Estimated cost-effectiveness of genetic vector control; References. (DOC) [file pone.0025384.s001.doc]

# Supporting information

**Models**

State variables are listed in Table 1 and parameters described in Table 2 (in the main text). Equations numbered 1 to 10 below are identical to those in the main text; supplementary equations are numbered S1 etc.

**Vector population dynamics model.** We modify Dye’s [1] deterministic, continuous time, time-delayed simulation model for the population dynamics of *Ae. aegypti*, which assumes a closed homogeneous population, with random mating, overlapping generations, 1:1 sex ratio and constant life history parameters. Designating the number of vectors, i.e. female adult mosquitoes, at time *t* as *F*(*t*), prior to the start of releases we have:

$\frac{dF}{dt}=PF(t-T)\exp\left\{-\alpha\left[2EF(t-T)\right]^{\beta}\right\}-\sigma F$ (S1)

Here, *T* is the generation time from egg to adult, 2*E* is the average daily egg production rate per female mosquito (Dye defined *E* as the average per adult), *P* is the per capita rate *E* reduced to reflect egg-to-adult mortality due to density-independent factors, $\alpha$ is related to the larval carrying capacity (number of breeding sites in the environment), $\beta$ is a measure of the strength of density-dependent competition between larvae, and σ is the adult mosquito per capita death rate per day (average adult lifespan,$\frac{1}{\sigma}$). The fixed time delay relates the rate of adult emergence of new females to the number of adult females (*F*) at the time those newly emerging vectors were laid as eggs (*T* days ago). This model reflects a form of “contest competition”; adults that survive high larval density have the same fitness as those that develop in lower densities.

Equation S1 has a stable equilibrium value $F^{*}$ for the number of vectors prior to RIDL release:

$F^{*}=\frac{\left[\frac{1}{\alpha}\log\left(\frac{P}{\sigma}\right)\right]^{\frac{1}{\beta}}}{2E}$ (S2)

The system can converge to this equilibrium either monotonically (weak density dependence, i.e., low $\beta<<1$) or with damped oscillations, or exhibit oscillations around the equilibrium (strong density dependence, high $\beta$). To allow for different size populations of vectors and human hosts, we define *k* as the average number of vectors (adult female mosquitoes) per host prior to RIDL control, and use this to calculate $\alpha$ (see Table 2). If the human host population is approximately constant ($\approx N_{0}$) over time, then by definition $F^{*}\approx kN_{0}$.

We modify equation S1 to incorporate the release, starting at time *t* = 0, of male mosquitoes that carry a dominant, late-acting, bisex-lethal genetic construct (“RIDL males”). All engineered released males are homozygous for the RIDL construct, which is assumed to be completely lethal, not sex-linked and imposes no fitness cost on released adult males (the construct is repressible, permitting mass-rearing). Females mate with wild or released males at random. We model a constant release policy, whereby mass-reared adult RIDL males are released in quantities that maintain their numbers at a fixed ratio (the “release ratio” *C*) to the equilibrium number of adult males or females in the wild population prior to initiation of the release program ($F^{*}$, equation S2), i.e. the total number of adult RIDL males $=CF^{*}$ for *t* > 0. The fraction of larvae that survive to adulthood is the ratio of wild type fathers to all adult males (wild type and released) $\frac{F(t-T)}{F(t-T)+CF^{*}}$. The engineered lethality acts after the effects of larval competition so all eggs ($2E\times F(t-T)$) contribute to the density-dependence term, regardless of their father’s genotype.

(1)

where $\tilde{C}=\left\{\begin{matrix} 0 \\ \\ C \end{matrix}\right.$ $\begin{matrix} t-T\leq0 \\ \\ t-T>0 \end{matrix}$

To maintain the number of RIDL males at the required constant value, $CF^{*}$ of them must be released instantaneously at $t=0$, and thereafter an average of $\sigma CF^{*}$ must be released daily to replace the losses from natural mortality. In practice, the initial release would likely be achieved by rapid build-up over a short period but we do not expect this simplification to have a significant effect on our conclusions. We ignore seasonality in vector dynamics; Wearing and Rohani [2] found that seasonality in vector recruitment is necessary to explain intra-annual variation in dengue incidence but that it has a lesser impact on inter-annual dengue dynamics, and we are interested primarily in assessing a control program running over several years.

**Epidemiological model.** As well as tracking the total number of vectors (equation 1) we use equations for the numbers of susceptible vectors (*X*) and vectors infectious with each serotype *i* (*Y_i_*). Vectors do not recover from infection [3]. The last expression in each equation is natural deaths from that class (we assume dengue has no effect on vector mortality as there is no evidence of any significant effect). We do not allow concurrent infections in either vectors or hosts. We assume no vertical transmission of dengue so all newly emerging vectors and all human new-borns are susceptible.

(2)

The first term on the right hand side is the emergence of new wild type adults, which is the same as in equation 1. The second term represents infections; mosquitoes bite at rate *b* and if susceptible (*X*), on biting an infectious host (*I_i_* or *I_ji_* among *N* hosts), a proportion (*c*) will become infected with serotype *i*. ADE may manifest as increased transmissibility from a host with a secondary infection (if $\zeta>1$), but we do not invoke this alternative scenario in our simulations (we keep $\zeta=1$). After a fixed time delay, the extrinsic incubation period ($\omega$), those vectors become infectious (*Y_i_*) with serotype *i* and remain infected for the rest of their lives.

$\frac{dY_{i}}{dt}=\left(\exp(-\sigma\omega)\right)\left(\frac{cbX(t-\omega)}{N(t-\omega)}\left(I_{i}(t-\omega)+\zeta I_{ji}(t-\omega)\right)\right)-\sigma Y_{i}$ $i,j=1,2$; $j\neq i$ (3)

The disease dynamics in the hosts are more complicated. The total number of hosts (*N*) will potentially grow or decline exponentially, although in our simulations we set the per capita birth rate ($\nu$) equal to the per capita death rate ($\mu$) so that the population would remain constant if there were no deaths due to DHF, and will be approximately constant if the proportion of hosts surviving a secondary dengue infection ($\rho$) is close to 1 (see equation 10 below).

$\frac{dS}{dt}=\nu N-\sum_{i=1}^{2} \left(ab\frac{F}{N}S\frac{Y_{i}}{F}\right)-\mu S=\nu N-\left(ab\frac{S(Y_{1}+Y_{2})}{N}\right)-\mu S$ (4)

Each susceptible host (*S*) is bitten at rate *b* by $F/N$ mosquitoes per host, of which $Y_{i}/F$ are infectious with serotype *i*, and a proportion *a* are successfully infected with the virus. After a fixed time delay, the intrinsic incubation period ($\tau$), they become infectious (*I_i_*), which lasts on average $1/\gamma$ days.

$\frac{dI_{i}}{dt}=\left(\exp(-\mu\tau)\right)ab\frac{S(t-\tau)Y_{i}(t-\tau)}{N(t-\tau)}-(\gamma+\mu)I_{i}$ $i=1,2$ (5)

The host retains lifelong immunity to the serotype of infection and has temporary cross-immunity to all other serotypes (*C_i_*, not to be confused with *C*, the release ratio) for an average period of $1/\psi$ days following the end of the infectious period. On challenge by a serotype not previously encountered cross-immunity is “classical”, i.e. no infection arises and no immunity is gained (as opposed to “clinical” cross-immunity where no symptoms arise and the virus is not transmitted onward but immunity is gained to that serotype). When the temporary cross-immunity has waned, the host is susceptible to infection by serotypes other than that from which s/he has recovered (*R_i_*).

$\frac{dC_{i}}{dt}=\gamma I_{i}-(\psi+\mu)C_{i}$ $i=1,2$ (6)

$\frac{dR_{i}}{dt}=\psi C_{i}-\left(\chi ab\frac{R_{i}Y_{j}}{N}\right)-\mu R_{i}$ $i,j=1,2$; $j\neq i$ (7)

ADE manifests both as increased susceptibility to a secondary infection ($\chi>1$),

$\frac{dI_{ij}}{dt}=\left(\exp(-\mu\tau)\right)\chi ab\frac{R_{i}(t-\tau)Y_{j}(t-\tau)}{N(t-\tau)}-(\gamma+\mu)I_{ij}$ $i,j=1,2;j\neq i$ (8)

and as a risk of DHF or DSS from secondary infection, with a proportion ($1-\rho$) dying at the end of the infectious period. On recovery from infection by the last remaining serotype (*R*), the host is immune to infection from all serotypes.

$\frac{dR}{dt}=\left(\rho\gamma(I_{12}+I_{21})\right)-\mu R$ (9)

$\frac{dN}{dt}=\nu N-\left((1-\rho)\gamma(I_{12}+I_{21})\right)-\mu N$ (10)

Together, the equations 1 to 10 form a system of 15 delay-differential equations representing the vector-pathogen-host dynamics. We run simulations in Matlab, incorporating the delay-differential equation solver dde23 [4]. Typically, we run the simulations for 200 to 250 years prior to beginning RIDL releases to allow the transient dynamics of the model to pass and settle to the expected pattern of epidemic cycles. Results here are presented after disregarding that initial period of atypical transient behavior.

Our model is not restricted to integer values. For the purposes of our simulations we regard the vector population (*F*) or the virus (the sum of all *I* values) as eliminated (locally) if it drops below 0.1 and remains there, and we label the earliest time from when that is true as the time to elimination.

**Sensitivity of combined epidemiological model to parameter values**

We varied one or sometimes two or three related parameter values, keeping all others unchanged, and assessed the qualitative effect on the behavior of the host-vector-pathogen system, including the average number of infectious cases arising in the 50 years before and after RIDL releases start. Significantly shorter mosquito lifespan ($\frac{1}{\sigma}=\frac{1}{3}$ days) prevented the disease from establishing at all. The disease could still become weakly endemic if the average vector lifespan ($\frac{1}{\sigma}=8.5$) was shorter than the fixed extrinsic incubation period ($\omega=10$ days), but at a lower prevalence; the benefit of genetic vector control would be less because there would be fewer “baseline cases” (i.e. average number of infectious cases in the 50 years before RIDL releases start) to avert, but this would be partly mitigated as the vector population could be eliminated more quickly (or with a lower release ratio) thus reducing the cost of doing so. A longer mosquito lifespan had the opposite effect, but our conservatively-chosen default value ($\frac{1}{\sigma}=14$) was close to that end of the range ($\frac{1}{\sigma}=15$) so the effect was not large. A significantly lower transmission success rate ($a=0.25$ or $c=0.2$) decreases the number of infections, epidemic peaks occur less frequently (roughly 7 or 9 years between peaks, respectively) , and the virus is eliminated more quickly; conversely a significantly higher transmission coefficient ($a=c=0.75$) causes a modest increase in numbers of cases. Higher transmission success would also reduce the entomological transmission threshold, so epidemics could arise with fewer vectors per host ($k=0.3$) in which case there would be fewer baseline cases (than with $k=2$), and fewer RIDL males would be needed for control. An extremely large number of vectors per host ($k=20$) by itself significantly increases the baseline cases and increases the frequency of epidemic cycles, and would take a proportionately greater number of RIDL males to achieve the release ratio. A lower or higher biting rate ($b=0.33$ or 1 per day), respectively decreases significantly (to 40%) or increases slightly the baseline cases and speeds or slows the epidemic cycles. A shorter or longer extrinsic incubation period ($\omega=7$ or 14 days) increases or reduces the baseline cases (by about 10%). If hosts recover very quickly ($\frac{1}{\gamma}=2$ days), very few mosquitoes acquire the infection, so the disease is barely able to persist. If hosts recover slowly ($\frac{1}{\gamma}=10$), more people are infected and stay infected for longer so the baseline cases increase dramatically (nearly double) and there is much greater potential benefit from the program. We conclude that the behavior of these model components is qualitatively consistent across the range of parameter values, and conforms to expectations about vector-borne disease dynamics.

**Determining basic reproduction number and entomological threshold (invasion analysis)**

For simplicity, we may assume the vector population is at equilibrium ($F^{*}$), and the host birth ($\nu$) and death ($\mu$) rates are equal and dengue-associated deaths are negligible ($\rho\approx1$) so that the host population (*N*) is constant. We can estimate the value of R_0_ inherent in our model from the dengue-naïve scenario (where all hosts and vectors are susceptible to dengue, $S\approx N$ and $X\approx F^{*}\approx kN$). Arguing from first principles, R_0_ is the number of mosquito infections that result directly (via hosts) from a single infectious vector mosquito entering the population. If that female mosquito survives the latent period (a proportion $e^{-\sigma\omega}$would), on average that vector will bite and successfully infect $\frac{ab}{\sigma}$human hosts during her lifespan. Each of those hosts that survives the latent period ($e^{-\mu\tau}$) will be bitten by and successfully infect $\frac{cbF}{N(\gamma+\mu)}$vectors on average while they remain infectious. Therefore

$R_{0}=\frac{e^{-(\sigma\omega+\mu\tau)}acb^{2}}{\sigma(\gamma+\mu)}\frac{F}{N}\approx\frac{e^{-(\sigma\omega+\mu\tau)}acb^{2}k}{\sigma(\gamma+\mu)}$ (S3, eq. 11)

$\approx2.97$ with default parameter values as in Table 2.

More formally, we use Dobson’s [5] method for multi-species pathogens, which uses the result that R_0_ can be computed as the dominant eigenvalue of a form of transmission matrix [6]. In this case of a vector-borne (two-species) disease, we modify the “who acquires infection from whom” (WAIFW) matrix, which contains the rates of infection between all possible combinations of species, to form a “next-generation” matrix, which contains those transmission rates multiplied by the duration of time for which an individual of the transmitting species is infectious. The next-generation matrix for our model is

$\left[\begin{matrix} 0 & \frac{e^{-\sigma\omega}\frac{cbX}{N}}{\sigma} \\ \frac{e^{-\mu\tau}\frac{abS}{N}}{\gamma+\mu} & 0 \end{matrix}\right]\approx\left[\begin{matrix} 0 & \frac{e^{-\sigma\omega}cbk}{\sigma} \\ \frac{e^{-\mu\tau}ab}{\gamma+\mu} & 0 \end{matrix}\right]$ (S4)

and solving for the dominant eigenvalue of matrix S4 gives the same result as equation S3.

With the assumption of constant host population, we can assess the conditions under which dengue (serotype 1, say) can invade naïve populations (where all hosts and vectors are susceptible to dengue, $S\approx N$ and $X\approx F^{*}\approx kN$) by expressing equations 3 and 5 in the form of an invasion matrix:

$\frac{d}{dt}\left[\begin{matrix} Y_{1} \\ I_{1} \end{matrix}\right]=\left[\begin{matrix} -\sigma& e^{-\sigma\omega}\frac{cb}{N}X(t-\omega) \\ e^{-\mu\tau}\frac{ab}{N}S(t-\tau) & -(\gamma+\mu) \end{matrix}\right]\left[\begin{matrix} Y_{1} \\ I_{1} \end{matrix}\right]\approx\left[\begin{matrix} -\sigma& e^{-\sigma\omega}cbk \\ e^{-\mu\tau}ab & -(\gamma+\mu) \end{matrix}\right]\left[\begin{matrix} Y_{1} \\ I_{1} \end{matrix}\right]$ (S5)

Expression S5 is equal to the zero matrix on the zero-growth isocline, at which the numbers of infectious hosts and vectors in almost entirely susceptible populations do not grow (or decline). Invasion analysis (“can dengue invade?”) is equivalent to linearizing the epidemiological model about the disease-free equilibrium (“is the disease-free state an unstable equilibrium?”). This threshold occurs when the determinant of the invasion matrix (S5) is equal to zero, i.e. where

$\sigma\left(\gamma+\mu\right)-e^{\sigma\omega+\mu\tau}cab^{2}k=0$ (S6)

Rearranging equation S6 gives the entomological threshold for disease transmission, i.e. the minimum number of vectors per host that are necessary to sustain the disease in a susceptible population, which we call $k_{d}$:

$k_{d}=\frac{\sigma\left(\gamma+\mu\right)e^{\sigma\omega+\mu\tau}}{cab^{2}}\approx0.67$ with default parameter values (Table 2) (S7, eq. 12)

The invasion threshold is where the basic reproduction number of dengue (R_0_) is equal to one [7].

### Validating basic reproduction number and transmission threshold

We estimated the value of R_0_, the basic reproduction number of any dengue serotype, at 2.97. This result compares plausibly with other estimates of dengue R_0_ obtained by different methods. Some older published estimates of 1.33 - 2, and a maximum of 2.5, are problematic [8]; for example one study used a formula developed for directly transmitted rather than vector-borne disease and another applied a formula that assumed a susceptible population to data from an area where dengue was endemic. For the 2005 outbreak in Singapore R_0_ was estimated to be ~1.89-2.23 (95% confidence interval: 1.15-3.00) using a single-phase Richards (logistic-type) model [9]. Analysis of age-stratified serotype-specific data collected in Thailand, using various rigorous statistical methods, gave strain-specific estimates of 1.38 – 8.47 across all models, and the best estimates put those R_0_ values in the range 4 to 6 [data from ref. 10, analyzed by ref. 11].

Our simulations identified an entomological threshold of about 0.67 to 0.68 adult females per person necessary to sustain dengue transmission in a susceptible population. This is consistent with Focks et al. [12], who used validated simulation models to estimate transmission thresholds in terms of the standing crop of pupae per person as a function of ambient air temperatures, initial immunity levels in the human population, and the size and frequency of viral introduction. Our result is within the range determined by those authors, converted using their ratios of standing crops of adult females to pupae per person at a range of temperatures. It is consistent with their results at an average temperature of about 28-29°C, which is reasonable for Thailand, for example, and the data supporting our parameter values.

### Estimated cost-effectiveness of genetic vector control

The cost-effectiveness of any disease control strategy is the cost of achieving a given outcome, such as the cost per case averted by the intervention. This could be judged by comparing with a benchmark level, with the equivalent figure for an alternative strategy, or with that for the current policy. Using more sophisticated measures, a hypothetical vaccination program against dengue has been claimed to be potentially very cost-effective [13]. It has been suggested that conventional mosquito adulticide spraying programs are not cost-effective [14], although a recent study of larviciding campaigns in Cambodia claims those can be very cost-effective [15]. For our study, we compare the projected costs per case averted by genetic vector control (based on costs of conventional SIT) with estimates of the average cost of an episode of illness.

**SIT costs.** A lot of the knowledge and expertise of designing, constructing and managing sterile insect production facilities has been transmitted by personal communication, internal memos, the involvement of the same governments and to some extent the same individual staff members in different programs, bilateral cooperation and international facilitation by the United Nations Food and Agriculture Organization and International Atomic Energy Agency (FAO/IAEA) joint program on nuclear techniques in food and agriculture, rather than through extensive literature, guides or published software [16]. We draw information from the available literature and various public sources on the costs and cost structures of existing and proposed SIT programs (Tables S1 and S2) and express costs in US$ inflated to 2008 values (see Methods).

Table S1 shows construction costs of SIT facilities. At smaller capacities, increasing size brings economies of scale, but large factories are built on a modular basis, so the cost relationship becomes more linear. We tried various possible fits of a power law $y=ax^{b}$and a rational function of the form $y=\frac{ax}{1+bx}$ to this data using non-linear least-squares regression. The proposed Australian facility to deal with a possible invasion of Old World screwworm was not approved, a decision that may have been influenced by the high cost – that data point significantly alters the regression curve – so it seems reasonable to remove this outlier from the data. At its current size, the El Pino Medfly facility dwarfs all others in the data set; that single data point does not significantly alter the fit of a power law curve, but it does alter the rational function. For the smaller scale of facility consistent with the number of mosquitoes to be released in accordance with our simulated results, the rational function with both outliers removed gives the best fit ($R^{2}=0.9786$):

SIT facility construction costs (US$ millions) = (S8)

where *x* is the weekly production capacity (millions of insects)

Table S2 shows budgeted or actual operational or production costs for SIT facilities, from a range of inconsistent sources. Some of these are purely production costs, and so exclude distribution or delivery costs; it is unclear what costs are included among the operational costs. There is no discernable pattern to this data to relate the cost per million insects to the production quantity. Screwworm facilities have higher costs, because of the difficulty of rearing a myiasis pest (flesh-eating maggots) and partly also because there is no sex separation and so both sexes must be reared in the final generation and released. Codling moths are also unsexed, but even allowing for that they are very much more expensive than the other SIT programs. We therefore exclude the codling moth data and use the mean cost (US$813 per million insects), and a range from the minimum (US$172 for mosquito SIT in India in 1971) to the mean plus standard error (US$813+819 = 1632).

**Costs averted through genetic vector control.** We use total cases, output from the simulation model, as our measure of the direct impact of the disease. The cost of dengue is estimated using a range of values for cost per case, based on published studies (Table S3). We also gather data on the per capita cost of conventional vector control methods (Table S4).

Table S3 shows the range of dengue costs reported in various studies, restated to 2008 US$. Where results are given separately, hospitalized cases typically cost roughly three times as much as non-hospitalized cases [17,18,19]. There is inconsistency between studies about which cases are included; many people infected with dengue are not hospitalized, so figures that include non-hospitalized cases (i.e. patients that are ill at home, many of whom will make outpatient visits to a hospital or clinic) are more appropriate for our purpose. Estimates that focus on direct costs and narrow (if any) indirect costs, or only on costs to the patient and ignore higher level costs to society, will be underestimates of the cost per case of illness – and therefore of the potential value of the benefit of reducing the number of cases.

The costs of vector control in several countries are shown in Table S4. More urbanized countries had higher spending (dengue is primarily an urban disease). The underlying studies were generally reporting on years when epidemics occurred. The reported actual or estimated expenditure varies by year and country, and they represent efforts that would have had differing levels of effectiveness; with few exceptions vector control has not achieved sustained control or prevention of dengue in recent decades. The data gathering protocols differ, but the amounts generally include both direct action such as larval source reduction or spraying adulticides, and related activities such as surveillance. For example, the figure reported for Malaysia was based on an estimated national cost in 2002 of US$5.8 million, split between the two major components of the program, 74% inspection and 24% fumigation. The figure for Cambodia is the weighted average of annual targeted larviciding campaigns in two regions reaching a population of 2.9 million, and comprises larvicide, operational costs, communications campaign and administrative costs.

This does not lead to a direct calculation of costs avoided because these costs of conventional vector control would not simply be entirely replaced by the costs of RIDL male releases. The SIT operational costs on which we base our cost estimates for the genetic vector control program also include some monitoring and surveillance, which would already be incurred and included within these estimates for conventional mosquito control. The RIDL male releases would likely be conducted as part of an integrated vector control program rather than as a replacement for the current practice. (Our simulation model does not include any adjustments to the vector population dynamics to reflect cessation of background level vector control, but neither does it include positive effects of such measures other than any that might be implicit in the pre-release equilibrium number of vectors per host.) With a successful RIDL release program the level of vector control activity would decrease, thus reducing costs. At first, fewer dengue cases occurring would trigger fewer reactive measures in the affected premises and neighborhood, and after local elimination of the vector has been confirmed, only surveillance would be needed for the purpose of watching for the return of the mosquito.

### References

1. Dye C (1984) Models for the population dynamics of the yellow fever mosquito, *Aedes aegypti*. Journal of Animal Ecology 53: 247-268.

2. Wearing HJ, Rohani P (2006) Ecological and immunological determinants of dengue epidemics. Proceedings of the National Academy of Sciences of the United States of America 103: 11802-11807. doi: 10.1073/pnas.0602960103.

3. Siler JF, Hall MW, Hitchens AP (1926) Dengue: its history, epidemiology, mechanism of transmission, etiology, clinical manifestations, immunity, and prevention. Philippine Journal of Science 29: 1-304.

4. Shampine LF, Thompson S (2001) Solving DDEs in MATLAB. Applied Numerical Mathematics 37: 441-458. doi: 10.1016/S0168-9274(00)00055-6

5. Dobson A (2004) Population dynamics of pathogens with multiple host species. The American Naturalist 164 S64-S78.

6. Diekmann O, Heesterbeek JAP, Metz JAJ (1990) On the definition and the computation of the basic reproduction ratio R_0_ in models for infectious diseases in heterogeneous populations. Journal of Mathematical Biology 28: 365-382. doi: 10.1007/BF00178324.

7. Fine PEM. Population biology of infectious diseases: The control of infectious disease. In: Anderson RM, May RM, editors; 1982 March 14-19; Berlin. Springer-Verlag. pp. 121-147.

8. Gubler DJ, Kuno G, editors (1997) Dengue and dengue hemorrhagic fever. Cambridge MA, USA: CABI Publishing.

9. Hsieh Y-H, Ma S (2009) Intervention Measures, Turning Point, and Reproduction Number for Dengue, Singapore, 2005. American Journal of Tropical Medicine and Hygiene 80: 66-71.

10. Sangkawibha N, Rojanasuphot S, Ahandrik S, Viriyapongse S, Jatanasen S, et al. (1984) Risk Factors in Dengue Shock Syndrome: A prospective epidemiologic study in Rayong, Thailand: I. the 1980 outbreak. American Journal of Epidemiology 120: 653-669.

11. Ferguson NM, Donnelly CA, Anderson RM (1999) Transmission dynamics and epidemiology of dengue: insights from age-stratified sero-prevalence surveys. Philosophical Transaction of the Royal Society London B 354: 757-768. doi: 10.1098/rstb.1999.0428.

12. Focks DA, Brenner RJ, Hayes J, Daniels E (2000) Transmission thresholds for dengue in terms of *Aedes aegypti* pupae per person with discussion of their utility in source reduction efforts. American Journal of Tropical Medicine and Hygiene 62: 11-18.

13. Shepard DS, Suaya JA, Halstead SB, Nathan MB, Gubler DJ, et al. (2004) Cost-effectiveness of a pediatric dengue vaccine. Vaccine 22: 1275-1280. doi: 10.1016/j.vaccine.2003.09.019.

14. World Bank (1993) World Development Report 1993: Investing in Health. New York: World Bank. 12183.

15. Suaya JA, Shepard DS, Chang M-S, Caram M, Hoyer S, et al. (2007) Cost-effectiveness of annual targeted larviciding campaigns in Cambodia against the dengue vector *Aedes aegypti*. Tropical Medicine & International Health 12: 1026-1036. doi: 10.1111/j.1365-3156.2007.01889.x.

16. IAEA (2008) Model Business Plan for a Sterile Insect Production Facility. Vienna: IAEA.

17. Anderson KB, Chunsuttiwat S, Nisalak A, Mammen MP, Libraty DH, et al. (2007) Burden of symptomatic dengue infection in children at primary school in Thailand: a prospective study. The Lancet 369: 1452-1459. doi: 10.1016/S0140-6736(07)60671-0

18. Armien B, Suaya JA, Quiroz E, Sah BK, Bayard V, et al. (2008) Clinical Characteristics and National Economic Cost of the 2005 Dengue Epidemic in Panama. American Journal of Tropical Medicine and Hygiene 79: 364-371.

19. Suaya JA, Shepard DS, Siqueira JB, Martelli CT, Lum LCS, et al. (2009) Cost of Dengue Cases in Eight Countries in the Americas and Asia: A Prospective Study. American Journal of Tropical Medicine and Hygiene 80: 846-855.

20. Singh KRP, Razdan RK (1975) Mass rearing of *Culex pipiens* *fatigans* Wied. under ambient conditions. WHO VBC 75.537.

21. Institut Haiwan (2002) Screw-worm fly laboratory. Available: <http://agrolink.moa.my/jph/ihk/swf1.htm>. Accessed: 2009 15 April.

22. Anaman KA, Atzeni MG, Mayer DG, Stuart MA (1994) Benefit-cost analysis of the use of sterile insect technique to eradicate screwworm fly in the event of an invasion of Australia. Preventive Veterinary Medicine 20: 79-98. doi: 10.1016/0167-5877(94)90109-0.

23. Ansari MA, Singh KRP, Brooks GD, Malhotra PR, Vaidyanathan Y (1975) The development of procedures for mass rearing of *Aedes* *aegypti* L. WHO VBC 75.560.

24. Dame DA (1985) Genetic control by sterilized mosquitoes. In: H. C. Chapman et al., editor. Biological control of mosquitoes. Fresno Bull. No. 6: American Mosquito Control Association, Inc. pp. 159-172.

25. Bailey DL, Lowe RE, Dame DA, Seawright JA (1980) Mass rearing the genetically altered macho strain of *Anopheles albimanus* Wiedemann. American Journal of Tropical Medicine and Hygiene 29: 141-149.

26. USDA (2006) Exotic Fruit Fly Strategic Plan FY 2006-2010. USDA, APHIS.

27. Parker A (2005) Mass-rearing for sterile insect release. In: Dyck VA, Hendrichs J, Robinson AS, editors. Sterile Insect Technique Principles and practice in area-wide integrated pest management. The Netherlands: Springer. pp. 209-232.

28. Von Allmen SD, Lopez-Correa RH, Woodall JP, Morens DM, Chiriboga J, et al. (1979) Epidemic Dengue Fever in Puerto Rico, 1977: a Cost Analysis. American Journal of Tropical Medicine and Hygiene 28: 1040-1044.

29. Kouri GP, Guzman MG, Bravo JR, Triana C (1989) Dengue haemorrhagic fever / dengue shock syndrome: lessons from the Cuban epidemic, 1981. Bulletin of the World Health Organization 67: 375-380.

30. Torres JR, Castro J (2007) The health and economic impact of dengue in Latin America. Cad Saúde Pública 23: S23-S31. doi: 10.1590/S0102-311X2007001300004

31. Añez G, Balza R, Valero N, Larreal Y (2006) Impacto económico del dengue y del dengue hemorrágico en el Estado de Zulia, Venezuela, 1997-2003. Revista Panamericana de Salud Pública 19: 314-320.

32. Clark DV, Mammen Jr MP, Nisalak A, Puthimethee V, Endy TP (2005) Economic impact of dengue fever/dengue hemorrhagic fever in Thailand at the family and population levels. American Journal of Tropical Medicine and Hygiene 72: 786-791.

33. Garg P, Nagpal J, Khairnar P, Seneviratne SL (2008) Economic burden of dengue infections in India. Transactions of the Royal Society of Tropical Medicine and Hygiene 102: 570-577. doi: 10.1016/j.trstmh.2008.02.015.

34. WHO-TDR (2007) Report of the Scientific Working Group on Dengue, 2006. Geneva, Switzerland: Special Programme for Research & Training in Tropical Diseases (TDR) / World Health Organization. TDR/SWG/08. 160 p.
