# Supplementary material for: A Model Framework to Estimate Impact and Cost of Genetics-Based Sterile Insect Methods for Dengue Vector Control
Source: PLoS One. 2011 Oct 5;6(10):e25384. doi: 10.1371/journal.pone.0025384 (PMC3187769; doi:10.1371/journal.pone.0025384)
Supplement: Table S1 — SIT facility construction costs. (DOC) [file pone.0025384.s002.doc]

**Table S1**

**SIT facility construction costs.**

| **Insect** | **Site** | **Construction from** | **Production capacity (millions sterile larvae / pupae per week)** | **Approx. (cumulative) construction cost US$ million** | **Approx. cost US$ million at 2008 prices** | **Ref. (see SI)** |
| --- | --- | --- | --- | --- | --- | --- |
| *Culex pipiens fatigans* | India - development | 1973 | 2.45 | 0.014 | 0.05 | [20] |
| Medfly | Argentina | 1991 | 200 | 4.5 | 6.50 | [16] |
| Medfly | Chile | 1992 | 50 | 2.3 | 3.24 | [16] |
| Medfly | El Pino stage 1 | 1996 | 500 | 4.2 | 5.45 | [16] |
| Medfly | Madeira | 1996 | 50 | 2.6 | 3.37 | [16] |
| Medfly | W Australia pilot | 1997 | 20 | 0.5 | 0.64 | [16] |
| Medfly | S Africa stage 1 | 1998 | 8 | 0.3 | 0.38 | [16] |
| Medfly | El Pino stage 1+2 | 1999 | 800 | 6.3 | 7.83 | [16] |
| Medfly | El Pino stage 1+2+3 | 2001 | 3500 | 21 | 25.00 | [16] |
| Medfly | Valencia, Spain | 2004 | 560 | 6.8 | 7.59 | [16] |
| Medfly | Israel | 2005 | 20 | 1.1 | 1.19 | [16] |
| Medfly | Bahia, Brazil | 2006 | 200 | 7.99 | 8.36 | [16] |
| Old World Screwworm | Australia / Malaysia | 1995 | 10 | 3 | 3.97 | [21] |
| Old World Screwworm | Australia - not built | (costed but not built) 1992 | 250 | 18.44 | 26.00 | [22] |
