# Supplementary material for: A Model Framework to Estimate Impact and Cost of Genetics-Based Sterile Insect Methods for Dengue Vector Control
Source: PLoS One. 2011 Oct 5;6(10):e25384. doi: 10.1371/journal.pone.0025384 (PMC3187769; doi:10.1371/journal.pone.0025384)
Supplement: Table S2 — SIT facility production or operational costs. (DOC) [file pone.0025384.s003.doc]

**Table S2**

**SIT facility production or operational costs.**

| **Insect** | **Site** | **Year of cost estimate** | **Production (millions sterile larvae / pupae per week)** | **Approx operational cost US$ per million released** | **Approx cost US$ per million at 2008 prices** | **Ref. (see SI)** |
| --- | --- | --- | --- | --- | --- | --- |
| *Aedes aegypti* | India - development | 1973 | 2.8 | 58 | 228 | [23] |
| *Culex pipiens fatigans* | India - development | 1973 | 2.45 | 50 | 196 | [20] |
| *Aedes aegypti / Culex quinquefasciatus* | India | 1971 | ? | 40 | 172 | [24] |
| *Anopheles albimanus* | El Salvador | 1979 | ? | 156 | 389 | [24,25] |
| Old World Screwworm | Australia / Malaysia | 1995 | 10 | 1084 | 1,433 | [21] |
| New World Screwworm | Jamaica | 1998 | 20 | 1700 | 2,141 | [16] |
| NW Screwworm | Mexico | 2001 | ? | 1900 | 2,262 | [16] |
| Mexfly | Texas, USA | 2004 | 55 | 410 | 458 | [26] |
| Medfly | Hawaii, USA | 2006 | 110 | 559 | 586 | [26] |
| Medfly | El Pino stage 1+2+3 | 2001 | 3500 | 220 | 262 | [16,27] |
| Codling moth | Canada | 2001 | 14 | 8500 | 10,119 | [16] |
| **All** |  |  |  |  | **1,659** | **Mean** |
|  |  |  |  |  | **2,912** | **Standard error** |
| **Excluding codling moth** |  |  |  |  | **813** | **Mean** |
|  |  |  |  |  | **819** | **Standard error** |
