# Supplementary material for: A Model Framework to Estimate Impact and Cost of Genetics-Based Sterile Insect Methods for Dengue Vector Control
Source: PLoS One. 2011 Oct 5;6(10):e25384. doi: 10.1371/journal.pone.0025384 (PMC3187769; doi:10.1371/journal.pone.0025384)
Supplement: Table S3 — Estimated costs per dengue case. (DOC) [file pone.0025384.s004.doc]

**Table S3**

**Estimated costs per dengue case.**

| **Country** | **Year** | **Nature of costs** | **Approx cost US$ million** | **Approx cost US$ million at 2008 prices** | **Approx cost per (ill / reported / clinically ill / hospitalized) case US$** | **Approx cost per case US$ at 2008 prices** | **Ref. (see SI)** |
| --- | --- | --- | --- | --- | --- | --- | --- |
| Puerto Rico | 1977 epidemic | direct costs (medical care & epidemic control measures), indirect costs (lost production workers/parents) | 6.0-15.6 | 17.3-44.9 | 26-31 | 75-89 | [28] |
| Cuba | 1981 epidemic | direct medical, patient social security pay, lost production, *Aedes* control | 103 | 215.3 | - | 625 | [29] |
|  |  | direct medical, patient social security pay, lost production (excludes vector control) | 60 | 125.4 | - | 364 | [29] |
| Nicaragua | 1994 epidemic | overall economic impact | 2.7 | 3.6 | 44 | 59 | [30] |
| Venezuela | 1997-2003 study | direct (medical), indirect (work absence patients/mothers) | 1.3 | 1.5 | - | 44 | [31] |
| Thailand | 1997 | direct patient costs | - | - | 23-67 | 29-85 | [32] |
|  | 2001 study | patient direct hospital costs, work days lost | - | - | 68 | 81 | [32] |
|  | 1994 | total direct costs of DHF | - | - | 257 | 347 | [32] |
|  | 1998-2002 study | primary school patient costs clinic/hospital, medication, family lost income | - | - | 17 | 19 | [17] |
| India | 2006 epidemic | median cost of treatment per hospitalized patient | - | - | 432 | 452 | [33] |
|  |  | economic burden (public & private healthcare, patient lost productivity) | 27.4 | 28.7 | - | - | [33] |
| Panama | 2005 epidemic | direct medical, non-direct medical, indirect – ambulatory / hospitalized | - | - | 332 / 1065 | 359 / 1152 | [18] |
| Eight countries Americas & Asia | 2005 study | direct medical, non-direct medical, indirect - all 8 countries: mean ±S.D. | - | - | 417±21 | 451±23 | [19] |
|  |  | - Americas | - | - | 733 | 793 | [19] |
|  |  | - Asia | - | - | 330 | 357 | [19] |
