# Supplementary material for: A Model Framework to Estimate Impact and Cost of Genetics-Based Sterile Insect Methods for Dengue Vector Control
Source: PLoS One. 2011 Oct 5;6(10):e25384. doi: 10.1371/journal.pone.0025384 (PMC3187769; doi:10.1371/journal.pone.0025384)
Supplement: Table S4 — Estimated per capita spending on vector control. (DOC) [file pone.0025384.s005.doc]

**Table S4**

**Estimated per capita spending on vector control.**

Sources: Supporting Information refs. 13,15,34

| **Country** | **Year** | **Cost per capita US$** | **Cost per capita US$ at 2008 prices** |
| --- | --- | --- | --- |
| Thailand | 1994 | 0.081 | 0.109 |
| Thailand | 1998 | 0.188 | 0.237 |
| Indonesia | 1998 | 0.015 | 0.019 |
| Singapore | 2000 | 2.400 | 2.925 |
| Malaysia | 2002 | 0.240 | 0.280 |
| Cambodia | 2001-2005 | 0.196 | 0.212 |
| 17 Caribbean Islands | 1990 | 0.140-8.490 | 0.210-12.733 |
|  |  | (median 1.340) | (median 2.010) |
| 14 Latin American countries | 1997 | 0.020-3.560 | 0.025-4.538 |
|  |  | (median 0.260) | (median 0.331) |
| **Mean** | | | 0.765 |
